# ADHD Rehabilitation through Video Gaming: A Systematic Review Using PRISMA Guidelines of the Current Findings and the Associated Risk of Bias

**DOI:** 10.3389/fpsyt.2015.00151

**Published:** 2015-10-22

**Authors:** Thiago Strahler Rivero, Lina Maria Herrera Nuñez, Emmy Uehara Pires, Orlando Francisco Amodeo Bueno

**Affiliations:** ^1^Grupo de Investigação em Memória Humana, Departamento de Psicobiologia, Universidade Federal de São Paulo, São Paulo, Brazil; ^2^Departamento de Psicologia, Universidad Libre, Cali, Colombia; ^3^Departamento de Psicologia, Universidade Federal Rural do Rio de Janeiro, Seropédica, Brazil

**Keywords:** neuropsychological rehabilitation, video game intervention, ADHD, real life, systematic review, PRISMA

## Abstract

Empirical research studies have highlighted the need to investigate whether video game can be useful as a tool within a neuropsychological rehabilitation program for attention deficit hyperactivity disorder (ADHD) patients. However, little is known about the possible gains that this kind of video game-based interventions can produce and even if these gains can be transferred to real life abilities. The present paper aims to uncover key information related to the use of video game in ADHD neuropsychological rehabilitation/intervention by focusing on its gains and its capability to transfer/generalize these gains to real life situation via a systematic review of the empirical literature. The PRISMA guidelines were adopted. Internet-based bibliographic searches were conducted via seven major electronic databases (i.e., PsycARTICLES, PsycINFO, Web of Science, Core Collection BIOSIS Citation Index, MEDLINE, SciELO Citation Index, and PubMed) to access studies examining the association between video game interventions in ADHD patients and behavioral and cognitive outcomes. A total of 14 empirical studies meeting the inclusion criteria were identified. The studies reported the attention, working memory, and the behavioral aspects as the main target of the intervention. Cognitive and behavioral gains were reported after the video game training (VGT). However, many bias related to the choice of outcome instruments, sampling and blindness of assessors, weaken the results power. Additional researches are important to clarify the effects and stability of the VGT programs, and an important effort should be made to construct better methods to assess improvements on everyday cognitive abilities and real world functioning.

## Introduction

### Rationale

Attention deficit hyperactivity disorder (ADHD) is the most common childhood behavioral disorder and typically first diagnosed during the school years (1, 2). It is characterized by inappropriate and persistent symptoms of inattention and/or hyperactivity/impulsivity that interfere in the quality of school, social and work functioning in daily life (3). ADHD individuals tend to have a wide spectrum of everyday problems, including self-regulation deficits, behavioral, social, and motivational symptoms ranging from heightened levels of aggression, poor sustained attention, cognitive flexibility deficits, shortened reward delay gradient, lower working memory span, difficulties on response inhibition, and temporal processing (4). ADHD can be treated using combined interventions, including stimulant medications, behavioral therapy, parent psychoeducation, and cognitive and social skills training (5). Several studies are focusing on the impact of rehabilitation programs for ADHD patients, some of them aiming at the restorative (drill and practice) treatment, others taking into consideration the everyday life impact of the training (functional approach).

New tools are being tested and implemented in the ADHD treatment as an approach to tap engagement, scaling, and adaptive training, such as virtual reality, neurofeedback, and video games. Games are now, more popular than ever in history, with numbers of players escalating to larger number every year (6). Although, many would argue that games can have detrimental effects, video games demonstrate a capacity to enhance cognitive tasks based on principles varying from probabilistic inference to focused attention (7). The use of health games has been a recent trend to serve as an ancillary mechanism in assisting individuals with cognitive problems (8–11). From a therapeutic perspective, this cognitive boost given by the video game could then be used in psychiatric and neurologic conditions treatment, where traditionally, the usual cognitive rehabilitation techniques are used to improve outcomes in conditions such as ADHD.

Traditional Computer-based training (CompBT) uses softwares designed to help patients improve cognitive functioning through sessions involving repetitive exercises. Few of them have video game elements that enhance the training effectiveness and the engagement with the treatment process; the latter directly influences drop rates and the overall efficiency of the training program (12).

Video game training (VGT) shows the same characteristics as the CompBT, plus the video game elements. Green and Bavelier (13) suggested that video game has a causal role in increasing the number of items kept in visual attention. Initial studies, investigating a single administration of video game in children and adolescents with ADHD, report that video game use promotes a state of great cognitive performance (by promoting cognitive feedback), increasing the activation state and excitement of participants (promoting enhanced motivational performance), increasing attention (14–16), and inhibitory responses (11, 17). The use of VGT in rehabilitation process employs video game elements (mechanisms, dynamics, and esthetics), which empower the learning and motivational process, and add CompBT characteristics, such as adaptive difficulty, database settings, and big data tools to work with much information generated via training.

Many studies have investigated the impact of video game use in behavior, habits, personality, and cognitive skills. Regarding cognition, studies indicate that healthy video game players outperform non-players in the performance of various tasks related to cognitive abilities, such as probabilistic inference, visual acuity, visual search capability in the face of distractors, visuospatial attention, divided attention, hand–eye coordination, time perception in milliseconds, and reaction time (18, 19).

In fact, in ADHD participants, some studies suggest that video game promotes an optimal cognitive performance by providing continuous feedback (20), improving attention (16), and inhibitory control (11), and heightening the activation/arousal state, which enhances motivational performance (11). It is important to investigate if these video game benefits can be employed to create new treatment protocols for use with ADHD patients. Some authors still support the idea that high motivational state promotes release of striatal dopamine (11, 14). Is it possible that this neurobiological state of increased motivation could increase the treatment engagement, thus decreasing the dropout rates? What are the challenges faced during the implementation of video games based treatment protocols? What kind of bias affects evaluation of using video games in the treatment of ADHD?

The current systematic review aims to bridge a gap in the literature related to the use of videogames in the treatment of ADHD. It employs Preferred Reporting Items for Systematic Reviews and Meta-Analyses (PRISMA) guidelines.

### Objectives

This review investigates and synthesizes a comprehensive set of cognitive gains resulting from controlled studies employing video games as an intervention for ADHD and the risks of bias involved in such evaluations. It also seeks to provide critical information on the assessment of generalizability of such cognitive gains to real world situations.

## Methods

This review was performed according to the PRISMA guidelines (21), thus providing a comprehensive framework which objectively assesses indicators of quality and risk of biases of included studies.

### Protocol and Registration

The protocol for this review was not previously registered.

### Eligibility Criteria

All original studies investigating the phenomenon of neuropsychological rehabilitation via video game technologies for ADHD and the resultant gains and transfer of such gains to real life situations were eligible for this systematic review. Further criteria adopted were: (i) publication date between 2000 and 2014, (ii) being an empirical study, (iii) written in English, Spanish or Portuguese language, (iv) published in a scholarly peer-reviewed journal, (v) conducted an intervention/rehabilitation/treatment for ADHD using video game technology, and (vi) a cognitive or cognitive-related construct objectively delimited as the aim of video game use. Additionally, studies were excluded from review if they were: (i) single-case report, (iii) single intervention session, (iv) insufficient data to be analyzed, and (v) non-cognitive or behavioral training (i.e., only motor training).

### Information Sources and Search

**Figure 1:**
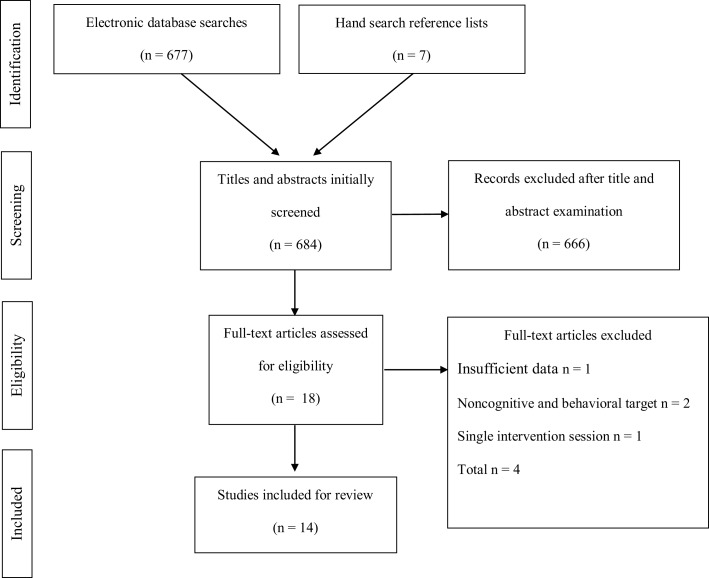
**PRISMA flow diagram of the study selection process**.

Studies were identified by searching relevant papers via EBSCO (2000-August, 2014-December), and included the following electronic databases: Academic Search Complete, PsycARTICLES, PsycINFO; via Web of Science (2000-January, 2014-December), which included Web of Science Core Collection, BIOSIS Citation Index, MEDLINE, SciELO Citation Index. In addition, there was an independent literature search on PubMed (2000-August, 2014-December). Finally, reference lists of retrieved studies were hand searched in order to identify any additional relevant studies. Key words and combination of key words were used to search the electronic databases and were organized following the Population Intervention Comparison Outcome (PICO) model (Figure 1). In this model, the search strategy can be organized based on the topics: population (P), intervention (I), control group (C), and outcome (O) and several searches in the aforementioned databases. Further, seven more studies retrieved from a reference in another study (22–25) were included.

(“attention deficit hyperactivity disorder” OR ADHD OR ADD OR “attention-deficit/hyperactivity disorder” OR “Attention Deficit Disorder with Hyperactivity” OR “Attention Deficit Hyperactivity Disorders” OR “Attention Deficit-Hyperactivity Disorders”) AND (video gam* OR computer gam* OR videogame OR game base OR game like OR game intervention) AND (treatment OR rehabilitation OR intervention) AND (cognitive functions OR follow-up assessment OR generalization OR transfer effects)

### Study Selection and Data Collection Processes

After performing the initial literature searches, each study title and abstract was screened for eligibility by the first author. Full text of all potentially relevant studies were subsequently retrieved and further examined for eligibility. The PRISMA flow diagram (see Figure 1) provides more detailed information regarding the selection process of studies. Information from the included studies was then analyzed and recorded in an electronic spreadsheet designed by the first author. Different types of data were extracted from each study including: (i) country in which the data were collected and participants’ characteristics, (ii) cognitive function intervention target, (iii) intervention protocol, (iv) risk of bias in individual studies, (v) follow-up assessment, (vi) Generalization and transfer effects, and (vii) limitation, among others.

The Cochrane Collaboration’s tool for assessing risk of bias was adopted to evaluate the risk of bias in individual studies (7, 26). The following risk of biases was analyzed: (i) *selection bias* (i.e., biased allocation to interventions due to inadequate generation of a randomized sequence, mainly addresses group allocation problems and outcome interpretation), (ii) *performance bias* (i.e., biases due to the knowledge of the allocated interventions by participants and personnel during the study), (iii) *detection bias* (i.e., biases due to knowledge of the allocated interventions by outcome assessors), (iv) *attrition bias* (i.e., biases due to the amount, nature or handling of incomplete outcome data), and (v) *reporting bias* (i.e., bias resulting from selective outcome reporting and/or not reporting relevant results outcomes that would have been expected to be reported [see for a complete description Higgins and Green (7, 26)].

As a complement to these types of biases mentioned, other two types were included (i) *sampling bias* (i.e., bias resulting in samples that do not represent the study population, mainly related to subject selection problem which undermines the generalization of results) and (ii) *measurement bias* (i.e., bias due to inappropriate use of scales or tests to measurement of ADHD symptoms and cognitive functions mainly related to non-validated criteria or inconsistent use).

## Results

### Study Selection

This review identified 677 studies (EBSCO *n* = 352; Web of Science *n* = 283; PubMed *n* = 42) after the initial search in the aforementioned electronic databases and seven (*n* = 7) retrieved from a list of references contained in another study was included in the identification process. The screening phase involved the examination of titles and abstracts of all studies identified. This process resulted in 666 studies being excluded, as they were deemed not suitable for the present review. A total of 358 studies were not exclusively dealing with ADHD patients; 211 did not employ video games for intervention; 97 were studies not focused on cognitive functions. Consequently, 18 studies were selected for the eligibility phase. Out of these, four studies were excluded mainly for (i) insufficient data to be analyzed (*n* = 1), (ii) non-cognitive or behavioral training (i.e., only motor training) (*n* = 2), and (iii) single intervention session (*n* = 1). Following this procedure, 14 empirical studies fully met the previously stipulated eligibility criteria for inclusion in the systematic review process (see Figure 1).

### Study Characteristics

More information about the essential methodological features and general characteristics of all reviewed studies can be found alongside Tables 1 and 2.

**Table 1:** **Main characteristics of the subjects in the Experimental Group (ExpG) of studies Pl**.

Study	*N*	Subject characteristics		Subtypes	Treat (%) ExpG	IQ/intellectual functions measures	Cognitive function intervention target	Intervention protocol	Main findings	Study limitations
		Age/mean (SD)	Male (%)							
van der Oord et al. (27)	40	8–12/IG: 10.00 (0.97)	82	EF: C; I; HI	66	WISC-III Estimated IQ IG: 101.11 (12.32) WLG: 103.36 (12.86)	Inhibition, cognitive flexibility and visualspatial and auditory WM	T/S: 40 min S/W: participant determine (total – 25) A/W: 5 weeks MP: NA	IG: improvement in parent-rated executive function and ADHD symptoms scales; improvement on inhibition and metacognition measures after training	Outcome measure: low power differences in teacher rating Other: technical difficulties as computer savings
Tamm et al. (28)	105	7–15/IG; 9.1 (1.2)	68	IG: C; I; not specified	65	WISC-IV Estimated IQ IG: 107.4 (11.5) WLG: 105.9 (13.3)	Attention	T/S: 30 min S/W: two sessions A/W: 8 weeks MP: NA	IG: Parents and clinicians reported fewer ADHD symptoms and attentional problems; improvement on sustained, selective, divided and alternating attention tasks.	Study design/method: limited sample size Gains and follow-up: necessary investigation on individual differences in response to treatment and to identify potential moderators
Chacko et al. (25, 29)	85	7–11/IG: 8.4 (1.4)	78	IG: C; I	27	WASI Estimated IQ IG: 104.2 (20.9) PG: 104.6 913.4)	Visualspatial and auditory WM	T/S: 30–45 min S/W: five sessions A/W: 5 weeks MP: NA	IG: improvement in verbal and non-verbal WM storage; no discernible gains in WM storage plus processing/manipulation	Study design/method: no waiting list condition. Gains and follow-up: conduction of longer follow-ups.
Lim et al. (30)	20	6–11/7.8 (1.4)	80	C; I	0	Exclusion criteria: known mental retardation (IQ 70 and below)	Attention and attention control	T/S: 30 min S/W: three sessions A/W: 8 weeks MP: once monthly booster for 3 consecutive months	All children: improvement in inattentive symptoms of ADHD; C: improvement in hyperactive-impulsive symptoms.	Study design/method: uncontrolled open-label trial Outcome measures: parents were not blinded completing rating scale; non-response rate from the children’s schoolteachers.
Johnstone et al. (31)	128	7–13/SW: 10.0 (2.1); SW + AM: 9.4 (2.2)	75	SW: C; I; HI SW + AM: C; I; HI	90	WASI Estimated IQ AD/HD group WL: 93.3 (13.8); SW: 94.0 (11.5); SW + AW: 92.7 (10.4) Control group WL: 106.3 (13.9); SW: 107.0 (13.6); SW + AW: 117.8 (13.0)	WM and Inhibition Control	T/S: 15–20 min S/W: participant determine (total – 25) A/W: 5 weeks MP: NA	IG: reduction in ADHD symptoms after training; Follow-up assessment: reduction at post-training was maintained 6 weeks after training.	Study design/method: no placebo training. Gains and follow-up: short and unique follow-up intervals; possible interaction with medication in training effects.
Steiner, et al. (32)	41	Not specified/12.4 (0.9)	51	Subtype not specified	60	Not specified	Attention and inhibitory control	T/S: 30 min S/W: two sessions A/W: 16 weeks MP: NA	Neurofeedback IG: reduction in behavioral and attentional symptoms rated by parents.	Study design/method: small sample size; different group characteristics.
Tucha et al. (33)	48	ATG: 10.8 (0.4)/PTG: 11.0 (0.6)	69	Subtype not specified	100	CFT 20 HG: 103.6 (1.3) ATG: 101.6 (2.9) PTG: 99.7 (2.6)	Attention	T/S: 45 min S/W: two sessions A/W: 8 weeks MP: NA	Attention training group: improvement on divided attention, vigilance and flexibility skills when compared to ADHD subjects in perception training group.	Gains and follow-up: neuropsychological assessment was not performed after the training.
Prins, et al. (9)	52	IG: 7–12/9.59 (1.12)	81	not specified	0	WISC-III Short version Substitution: IG: 9.0 (3.2); PG: 9.0 (2.5) Block design: IG: 10.74 (3.79); PG: 9.83 (2.96) Vocabulary: IG 11.85 (3.39); PG: (10.83 (2.93)	WM	T/S: 35 min S/W: 1 session A/W: 3 weeks MP: NA	WM training group: improvement in training performance, WM task and motivation at post-training.	Study design/method: did not control game elements and difficulty level; different group characteristics Gains and follow-up: no information about the stability of the effects is available; no follow-up assessments were conducted
Holmes et al. (34)	25	^a^8–11/9.9 (0.11)	84	C	100	WASI Verbal IQ Pre-training similarities 48.72 (10.27) Vocabulary 39.64 (11.19) WASI Perfor. IQ Block design: 48.44 (12.74) Matrix reasoning: 41.88 (12.86)	Visualspatial and Auditory WM	T/P: 35 min S/W: participant determine (total – 20–25) A/W: 6–10 weeks MP: NA	WM training: gains in all components of WM and STM (verbal and spatial) across untrained tasks. Training gains associated with the central executive: persisted over a 6-month period.	Study design/method: absence of comparison conditions control
Beck, et al. (35)	52	^a^7–17/11.75 (not specified)	69	C; I	61	Not specified	Visualspatial and Auditory WM	T/S: 30–40 min S/W: participant determine (total – 25) A/W: 6 weeks MP: NA	IG: reduction in inattention symptoms; improvement in initiation, planning/organization, and WM rated by their parents	Gains and follow-up: conduction of longer follow-ups; study training effects in other populations
Lim, et al. (36)	16	IG: 7–12/8.6 (1.4)	81	IG: C; I	0	Exclusion criteria: known mental retardation (IQ 70 and below)	Attention and concentration	T/S: 30 min S/W: two sessions A/W: 10 weeks MP: NA	IG and CG: improvement in hyperactive-impulsive symptoms. IG: improvement in inattentive scores (but did not reach statistical significance).	Study design/method: small sample size Procedure: frequent clinic visits
Shalev et al. (37)	36	6–13/IG: 9.1 (not specified)	83	IG: C; I	0	Not specified	Attention	T/S: 60 min S/W: two sessions A/W: 8 weeks MP: NA	IG: reduction of reported inattentiveness by parents; improvement on non-trained measures of reading comprehension and passage copying.	Outcome measure: no objective attentional measure in pretest; no teachers ratings Gains and follow-up: no follow-up assessments were conducted
Klingberg et al. (38)	53	IG: 7–12/9.9 (1.3)	83	IG: C; I	0	Exclusion criteria: IQ < 80 (based on an IQ test or the physician’s clinical impression and school history)	Visualspatial and auditory WM	T/S: 40 min S/W: participant determine (total – 25) A/W: 5–6 weeks MP: NA	ADHD: reduction in symptoms of inattention and hyperactivity/impulsivity rated by parents, both post-intervention and at follow-up; effect in visualspatial WM tests and for secondary outcome tasks	Study design/method: small sample size Outcome measure: non-standardized psychiatric interview was not performed Gains and follow-up: need of study training effects in other populations; effect of combining medication with training was not investigated; additional follow-up measurements is necessary
Klingberg et al. (39)	14	IG: 7–12/11 (2)	79	Subtype not specified	43	Before training RCPM IG:26.4 (1.2) PG: (28.7 (0.8) RAPM PG: 12.25 (0.25)	Visualspatial and auditory WM	T/S: 25 min S/W: not specified A/W: 5–6 weeks MP: NA	IG: improvement in outcome measures – trained WM, span board, Raven’s progressive matrices, stroop accuracy, and number of head movements	Gains and follow-up: no effects everyday life for children with ADHD; no investigation of the durability of the training effects

*ADHD, attention deficit hyperactivity disorder; IG, intervention group; PG, placebo group; HG, healthy group; ATG, attention training group; PTG, perception training group; EF, executive function training; WLC, waiting list control; SW, software; SW + AM, software with attention monitoring C, combined subtype; I, inattentive subtype; HI, hyperactive/Impulsive subtype; WM, working memory; STW, short-term memory; T/P, time per session; S/W, sessions per week; A/W, amount of weeks; MP, maintenance phase; WISC, Wechsler Intelligence Scale for Children; WASI, Wechsler abbreviated scale of intelligence; RCPM, Raven’s colored progressive matrices; RAPM, Raven’s advanced progressive matrices; CFT 20, Culture Fair Intelligence Test Scale; Treat (%), percentage of subjects in medication; ExpG, experimental group; ContG, control group*.

*^a^The author gives only the data from the total sample, does not divide the Mean (M) or SD between Experimental group and Control Group*.

**Table 2:** **ADHD neuropsychological rehabilitation through video game interventions**.

Supporting research	Type of study and design	Country of origin	Video game type	Independent outcome measures		Post training assessment	Follow-up findings	Generalization and transfer effects findings
				Rating instruments	Cognitive tests			
van der Oord et al. (27)	RCT	Netherlands	3D adventure mini games	BRIEF, DBDRS (parent and teacher-rated)	No cognitive tests	6 Weeks of the final training day	9 Weeks – maintenance of the training protocols gains	Not evaluated
Tamm et al. (28)	RCT	United States of America	Board and computadorized mini games	SNAP-IV. BASC-II, C GI, ATTC, BRIEF (parent and teacher-rated)	TEA-Ch, WISC-IV, WJ-III, D-KEFS, Quotient ADHD system	12 Weeks after baseline	NA	Participants rated themselves as having significantly improved ability to focus their attention and shift their attention
Chacko et al. (25, 29)	RCT	United States of America	3D adventure mini games	DBD (parent and Teacher-rated)	AWMA, WRAT4-PMV, CPT	3 Weeks after the final training day	NA	Transfer to a non-trained skills (Dot Matrix and Digital Recall)
Lim et al. (30)	CT	Singapore	3D adventure mini games	ARS-IV (parent-rated)	No cognitive tests	After the final training day – week 8	Three once monthly booster training sessions Maintenance of the training protocols gains	Not evaluated
Johnstone et al. (31)	RCT	Australia	3D adventure mini games	BRS (parent-rated and other significant adult)	Go no go, Oddball task, Flanker task, Couting span, Digit-span	30–35 days after the pre-training session	6 Weeks – maintenance of the training protocols gains	Not evaluated
Steiner et al. (32)	RCT	United States of America	Simulator (flying)	CRS-R, BRIEF, BASC-2 (parent and teacher-rated)	IVA-CPT	1 Month after the intervention	NA	Not structured parents reports: improvement on focus skill, improved organizational and study skills, including ability to start the project and finish it.
Tucha et al. (33)	RCT	Germany	3D adventure mini games	No rating scales	Computerized neuropsychological tasks of attention	After the final training day – Week 8	NA	Transfer to a non-trained skills (flexibility
Prins et al. (9)	RCT	Netherlands	3D adventure mini games	No rating scales	Corsi block Tapping Test	Week 3	NA	Not evaluated
Holmes et al. (34)	RCT	United Kingdom	3D adventure mini games	No rating scales	AWMA, WASI	After the final training day	6 Months – maintenance of the training protocols gains	Not evaluated
Beck, et al. (35)	RCT	United States of America	3D adventure mini games	CRS-R, BRIEF (parent and teacher-rated)	No cognitive tests	Parent: 1 and 4 months after their child completed the intervention Teacher:1 month after the intervention and 4 months after the intervention for the experimental group	4 Months – maintenance of the training protocols gains	Not evaluated
Lim et al. (36)	CT	Singapore	Simulator (racing)	ARS-IV (parent and teacher-rated)	No cognitive tests	After the final training day – Week 10	NA	No result was shown
Shalev et al. (37)	RCT	United Kingdom	Mini games	PRS – Parents Rating Scale (Parent-rated)	Passage copying, Math exercises, Reading comprehension	Within 2 weeks of completing the treatment	NA	Not evaluated
Klingberg et al. (38)	RCT	Sweden	3D adventure mini games		The span-board task, digit-span, The stroop interference task, Raven’s colored progressive matrices	5–6 Weeks after the baseline	3 Months – maintenance of the training protocols gains	Transfer to a non-trained skills (span board)
Klingberg et al. (39)	RCT	Sweden	3D adventure mini games	No rating scales	Visuospatial WM task, span board, stroop task, Raven’s colored progressive matrices and choice reaction time task	5–6 Weeks after the baseline	NA	Transfer to a non-trained skills (span board and reasoning skills)

*RCT, Randomized Clinical Trial; CT, Clinical Trial; ARS-IV, ADHD rating scale IV; PRS, Parents Rating Scale; CRS-R, Conners’ Rating Scales–Revised, BRIEF, Behavior rating inventory of executive function; BASC-2, Behavior Assessment Scale for Children; IVA-CPT, Integrated Visual and Auditory and Continuous Performance Test; BRS, Behavior Rating Scale; SNAP-IV, DSM-IV ADHD Rating Scale; CGI, Clinical Global Impressions, ATTC, Attentional Control Scale, TEA-Ch, Test of everyday attention for children, WISC-IV, Wechsler Intelligence Scale for Children, WJ-III, Woodcock Johnson Tests of Achievement, D-KEFS, Delis–Kaplan executive function system, DBDRS, Disruptive Behavior Disorders Rating Scale; AWMA, Automated working memory assessment; WRAT4-PMV, Wide range achievement test 4 – Progress monitoring version; DBD, Disruptive Behavior Disorders rating scale; WASI, Wechsler abbreviated scale of intelligence*.

#### Country in Which the Data Were Collected

Four studies were from the United States (27–30, 32, 35–37), two were from The Netherlands (9, 27), two from Singapore (30, 36), two from United Kingdom (34, 37), two from Sweden (38, 39), one from Germany (33), and another one from Australia (31).

#### Participants

The studies reviewed included 715 participants. In terms of gender distribution (1), the vast majority of the studies reviewed recruited more male participants (*n* = 543, 75.92%) than female participants (*n* = 172, 24.08%). Not surprisingly, all of the reviewed studies explicitly included children samples.

Furthermore, all studies enrolled ADHD participants, eight studies included inattentive and combined ADHD subtypes (27–30, 35–38) and one study included hyperactive subtype alongside with combined and inattentive (31). Other studies did not specify the participants ADHD subtype (9, 32–34, 39)

Stimulant medication is a common confounder in video game treatment studies. In a clinical study, before starting the rehabilitation process, it is important to know the effects of the medication use in the participant and how long these effects have been occurring. This caution ensures the reliability of the observed effects and results. Five studies recruited medication free participants. Six studies had nearly 75% of its participants on medications. In three studies, over 90% of the subjects were on medications.

Finally, the intelligence of the children and adolescents with ADHD is relevant information for the pre-assessment and intervention. All selected studies included a measure of intelligence quotient (IQ) as an exclusion criterion [such as Wechsler Intelligence Scale for Children (WISC), Wechsler Abbreviated Scale of Intelligence (WASI), Raven’s Matrices], but three did not provide this information (32, 35, 37)

#### Operationalization of Cognitive Treatment Targets

Operationalization of a cognitive function comprises describing how a treatment target was objectively defined and characterized in the reviewed studies. Six studies (28, 30, 32, 33, 36, 37) targeted several attention function such as selective, sustained, orienting, divided, vigilance, and alternating. Eight studies had their video game built focusing on working memory (9, 27, 28, 31, 34, 35, 38, 39) The other three studies (27, 31, 32) had inhibitory control abilities as their main treatment target.

#### Operationalization of Video Games Genre

Operationalization of a video game genres aims to describe the game’s mechanics, dynamics, and esthetics employed in each VGT (40). Ten studies (9, 27, 29–31, 34, 35) employed 3D Adventure mini games, this kind of games employs elements of puzzles, exploring, discovering, and mini games related to brain challenges. Two games (32, 36) were constructed as simulators, which depict real world situations. Furthermore, one game (28) was a mixed board and computerized brain challenge mini games and one game used simple mini games (37).

#### Methodological Features of Studies

Concerning studies’ key methodological features, all reviewed studies were of empirical nature and the great majority of reviewed studies were randomized controlled trial (RCT) (12 studies from 14), often considered the gold standard for a clinical trial. However, Lim et al. (36) used a controlled trial without randomization and Lim et al. (30), an uncontrolled open-label trial, as described in their articles.

Ten studies used rating scales for collecting data, but four of them used rating scales only (27, 30, 35, 36), one study (37) associated rating scales and formal education evaluation, four studies (27, 31, 32, 38) used rating scales and cognitive tests methods and one study employed the three above cited tools (29) to assess participants. Additionally, four studies employed only cognitive tests as outcome evaluation tools (9, 33, 34, 39).

With respect to rating scales employed in the outcome, three studies employed parent evaluation only (27, 30, 37), five studies used Parent and Teacher rating (29, 32, 35, 36, 38), one study (31) employed parents and other significant adult and one study associated ratings from parents, teachers, clinicians, and the participants (28). Moreover, four studies (9, 33, 34, 39) did not use any type of external assessor, only the participant’s results in cognitive tests.

#### Video Game Protocols Characteristics and Effects of Video Game Intervention on ADHD Participants

Four distinct characteristics were analyzed in relation to video game protocols, amount of time per training session, amount of sessions per week, amount of weeks and if the study employed maintenance phase.

Twelve studies employed a minimum of 30 min of video game playing per training session; only two studies trained their participants for less than 30 min (31, 39).

The two most common weekly training regimen were: two sessions per week (28, 32, 33, 36, 37) and a free to play style where the participants can choose how many times they can play per week from a total of 25 sessions (27, 31, 34, 35, 38). Moreover, one study applied three sessions per week (30), another one applied five times a week (29), and the last one has not specified the number of times per week (39).

The minimum number of training weeks was 3 (9). Six studies trained their participants during 5–6 weeks (27, 29, 31, 35, 38, 39), five studies employed an 8-week regimen of training (28, 30, 33, 36, 37), one study used 6–10 weeks regimen (34) while another had 16 weeks of intervention (32).

The last characteristic that was evaluated related to the training protocols was whether the studies had a maintenance phase, where the participants came back to the training center to have one or more training sessions after the study was concluded. Only one study employed monthly booster sessions. This was for 3 months (30).

In terms of the effects found of video game intervention on ADHD participants, ten studies used scales to measure the outcomes of their intervention. Five studies (27, 28, 30, 32, 38) found a reduction on both inattentive and hyperactive-impulsive symptoms. Two studies described a reduction in inattentive symptoms only (35, 37) and another one found a reduction on hyperactive-impulsive symptoms only (36). In relation to scales, studies reported enhancement in abilities related to inhibition and metacognition (27), initiation, planning/organization and working memory (35), attentional control (28), motivation on task (9), and schooling skills, such as reading comprehension and passage copying (37).

Seven studies employed more than scales in order to evaluate their students. These studies used standardized tests as outcome measures after video game intervention. Five studies (9, 29, 34, 38, 39) found a better performance on working memory skills, like visuospatial and verbal working memory. Two studies (28, 33) reported enhanced performance on attention skills such as selective, sustained, divided, vigilance, set-shifting, and response speed. Three studies found that video game intervention promoted better scores for ADHD participants in executive functions, such as flexibility skills (33) and response inhibition (38, 39). Besides, these last two studies found that participants had a better performance on complex reasoning tasks. Only one study evaluated short-term memory (verbal and spatial), and the results support for a better performance after the VGT (34).

##### Follow-Up Assessment

Seven studies (27, 29–31, 34, 35, 38) had a follow-up session to assess the maintenance of the gains related to the video game intervention. The time varied from as early as 3 weeks (29) to 6 months later (34) in the maximum. All the seven studies, except for one (29) showed maintenance in the training gains in each follow-up assessment.

##### Transfer and Generalization Effects

Seven studies did not evaluate transfer effects (9, 27, 30, 31, 34, 35, 37). Four studies related transfer to non-trained skills such as flexibility (33) and working memory (29, 34, 38, 39). Two studies used reports from parents (32) and participants (28) account for improvements on attentional, organizational, and study skills. One study (36) used mathematics and English exercise worksheets as a measure of skill transfer. However, the results were not presented in the study.

Other than some study skills as stated above, no other life skills were evaluated for generalization in any of the studies.

### Limitations Assessments

This review provides a systematic overview and important guidelines for future research in ADHD neuropsychological intervention using video games. However, further studies are necessary to clarify certain aspects about the study design and method, outcome measures, follow-ups investigations and transfer effects of the training.

For example, regarding the difficulties about the study design and method, two mentioned the need for well-designed RCTs (30, 36), three other studies did not adopt a wait-list, a placebo and a control training condition (29, 31, 34), two did not control game elements, difficulty level and medication (9, 31), and one used a non-adaptive training (27). More specifically, five studies highlighted that the small sample size could have affected the statistical power of the trial (28, 32, 36, 38), other two studies had different group characteristics, more boys (9) and medium-to-high socioeconomic status (32).

The assessments and outcome measures have an important role in gains and effectiveness of the intervention. Indeed, two studies had non-response rate from the children’s teachers (28, 30), one study did not have teachers’ ratings (37), another one, the parents were not blind (30) and one study did not apply a neuropsychological assessment after the training (33), which makes the results interpretation and the evaluation of the training program efficacy difficult. Moreover, variability on some outcome measures (27) and low power differences in teachers’ ratings (30) were also discussed.

In intervention studies, one of the most significant results is the effect and the benefits of the training programs. However, some studies did not obtain information about training gains for all cognitive assessments (34), the effects in everyday life (39), the effect of combining medication with training (38), the training effects in other populations (35, 38), the stability or durability of the effects (9, 39).

Follow-up assessments are essential to assess whether gains attributed to intervention are maintained over time. Despite the importance of performing this kind of assessment, such analyses were not conducted (9, 37). On the other hand, four studies emphasized the necessity for longer follow-up intervals (29, 31, 35, 38).

Other difficulties mentioned include the potentially demotivating effect of high frequency of clinic visits (36) and the technical issues such as saving data on computers that can impact on validation of the training modules (27).

### Risk of Bias in Individual Studies

A general overview and summary of possible risks of bias across all reviewed studies is presented in Table 3. However, among all studies analyzed, only two studies (29, 38) did not meet any type of bias.

**Table 3:** **Assessment of risk of bias in individual studies**.

Study	Selection bias	Performance bias	Detection bias	Attrition bias	Reporting bias	Other bias	
						Sampling bias	Measurement bias
van der Oord et al. (27)	–	‘+	‘+	–	–	–	‘+
Tamm et al. (28)	?	‘+	‘+	–	–	–	–
Chacko et al. (25, 29)	–	–	–	–	–	–	–
Lim et al. (30)	–	‘+	‘+	–	–	‘+	‘+
Johnstone et al. (31)	‘+	–	?	‘+	‘+	–	–
Steiner et al. (32)	–	‘+	‘+	‘+	–	?	–
Tucha et al. (33)	?	?	–	–	–	‘+	‘+
Prins et al. (9)	‘+	‘+	–	–	–	‘+	‘+
Holmes et al. (34)	–	?	–	–	–	–	‘+
Beck et al. (35)	‘+	‘+	‘+	–	–	–	‘+
Lim et al. (36)	–	‘+	‘+	–	+	‘+	‘+
Shalev et al. (37)	–	‘+	‘+	–	‘+	–	‘+
Klingberg et al. (38)	–	–	–	–	–	–	–
Klingberg et al. (39)	–	–	–	?	–	‘+	‘+

*‘+high risk of bias; –low risk of bias; ^?^unclear risk of bias*.

In terms of selection bias, three studies (9, 31, 35) were rated as high risk due to increased likelihood of bias resulting from (i) non-random component in the sequence generation process and/or (ii) non-random allocation of participants that involved judgment or other non-random method of categorization of participants (e.g., results of test scores assessing ADHD). Potential performance bias was found to be of high risk across eight studies (9, 27, 28, 30, 32, 35–37) as in these studies, blinding of key study participants and personnel were not achieved, thus representing potential sources of biases at the outcome levels. Detection bias potentially posed high risk in eight studies (32, 33, 35, 37) due to knowledge of the allocated interventions by outcome assessors. Regarding attrition bias, only three studies (31, 32) were rated as high risk for potential attrition bias due to the amount or unclear nature of handling of the missing data. Reporting bias was rated high risk in three studies (31, 36, 37) as some key variables that would have been expected to be reported were not.

Assessment of other sources of biases involved the examination of sampling bias and measurement bias. Sampling bias was rated as high risk in five studies (9, 30, 33, 36, 39) due to (i) widespread use of self-selected samples, (ii) lack of probability-sampling techniques, and/or (iii) recruitment of male-only samples. In addition, measurement bias was judged as high risk in the vast majority of studies (9, 27, 33, 35, 39) due to (i) inconsistent conceptualization of cognitive target, (ii) inconsistent measurement of the outcomes, and/or (iii) inconsistent selection of tools and instruments to evaluate outcome.

## Discussion

The present review aimed to identify relevant empirical evidence for the effect of video games interventions on cognitive training for ADHD patients.

Despite the trend that proposes that video game shows causal effects for cognition training on healthy subjects [e.g., visual attention (30)], it is still quite challenging to assess the impact of video games in the rehabilitation process. Several confounders such as family’s perception, teachers’ perception, stimulant drugs use, IQ level, assessors intervention, game genre, participant’s gender, game’s mechanics–dynamics–esthetics and transfer/generalization evaluation tools are some of these challenges.

Furthermore, there is still controversy about how to measure generalization and transfer effects to patient’s daily life. Quite a few of the analyzed studies claim to have produced generalization to near non-trained skills, however, the tasks employed as generalization measure are very similar to the video game employed in the training (29, 33, 38, 39) [to further this discussion, see Green and Bavelier (41)]. Other studies propose that their game transfer gains to real life abilities; yet the instruments (such as verbal report from parents and participants) are not appropriate to this kind of measurement (28, 32). It is essential to the advance of the video game research field that the results and findings are analyzed in the light of a risk bias assessment to assess the strength of our cumulative evidence in the area.

Even with a steady growth in the number of “games” designed to reduce cognitive deficits, most training softwares are designed for one aspect only and their evaluation falls into what we refer to as the “dual problem,” failing to achieve their rehabilitation aims: (1) games focused on training cognitive skills (such as working memory) but using only symptoms scales to evaluate outcomes and treatment success; and (2) games focused on training cognitive skills (such as working memory) but using only cognitive/neuropsychological testing to assess outcomes and treatment success. This dual problem needs to be overcome by using assessments tailored to the patient’s own problems in addition to symptoms scales and neuropsychological testing.

Concerned by this issue, some studies have ecologically valid scales such as Behavior Rating Inventory of Executive Function (BRIEF in order to get closer to measuring aspects related to the real life of the participants. Bisoglio et al. (42) proposed an important advance to VGT field would be the implementation of individualized baseline measures to be subsequently used as treatment response predictors. These kinds of measures exist and have been used since 1968 for fields such as neuropsychological rehabilitation and occupational therapy. These are instruments such as the Goal Attainment Scale (GAS), (43). The GAS is an individualized measure of change that involves defining a set of goals for each research participants and specifying a unique range of outcomes which reflects the patient’s real life struggles (44). GAS has been widely used with success for several areas related to rehabilitation, including neuropsychological rehabilitation of Sensory Modulation Disorder (45), ADHD (46), Metacognition on mental health (47), Acquired Brain Injury (48), and more [for a revision on its use, see Krasny-Pacini et al. (49)]

### Country in Which the Data Were Collected and Participant’s Characteristics

In terms of geographic dispersion, most studies (i.e., 12) were conducted either in the United States and/or European context, while four studies were from Asian/Australian regions. Across all reviewed studies, participants’ patterns suggested that the studies generally recruited more (i) male participants than female, (ii) children and adolescents’ samples than adults and/or elderly ones, and (iii) combined ADHD subtypes rather than inattentive and hyperactive-impulsive populations. Given the present findings, it is important that future research on video game interventions for ADHD include: (i) a more representative sample ADHD subtypes, considering the ADHD distribution percentages, (ii) Recruit subjects from across the life span, (iii) Include subjects from across all cultures. On the other hand, the relative increase of participants being recruited in VGT/treatment studies is, perhaps, a positive aspect.

### Operationalization Cognitive Treatment Targets

In terms of outcome measures, assessors and tools employed for rating and cognitive evaluation, attention should be paid by researchers to the way that cognitive targets are operationalized for posterior treatment and assessment in their research.

Evidence from the present review suggests that the studies analyzed measured attention, working memory and inhibition control. With regard to the attention construct, for instance, measured by a more “pure” and construct-based test, such as the Continuous Performance Test (CPT) is different from the Behaviour Assessment Scale for Children (BASC) and can lead to misinterpretation of the results when compared. Other important point here is that the outcome that the researchers are looking for is change in patient daily life problems, but again, as pointed out by Towne et al. (50), the instruments employed to evaluate this outcome did not resemble the real-world demands. In terms of instruments to evaluate outcome, 93% used symptom and behavioral scales, 79% of the studies employed psychometric tests, but only 14% of the studies utilized formal measures to evaluate academic gains after VGT. However, only one of the games had elements that resemble problems evaluated by the symptoms and behavioral scales (28).

Almost all studies employ symptoms and behavioral scales as measures of changes in daily life problems, thus expecting that instruments that mainly remain on parents’ and teacher’s perception will be sufficiently sensitive to track changes based on cognitive training only. Boot et al. (8) emphasizes that if the baseline measures for transfer effect are inadequate, it will be hard to evaluate the findings and to understand if the near and far transfer effects exist or are methodological noise.

Two of the present researches used only parents to evaluate the outcomes, seven, employed parents and teachers and one assessed parents and other significant adults. Only one research (28) reported that a rating filled by the participants about their perception of skill gains after the VGT was employed. It is imperative not to limit to the perceptions of one assessor only (not to incur in a single responder bias, see Fergunson et al. (51) and to refine and produce a more sensitive change measurement, including social perception of change, participant’s perception of change, structured changes expected after the training, along with cognitive changes measured via neuropsychological tests.

### Video Game Characteristics and Intervention Protocol

Video game can provide different types of involvement and experiences. One of the most important is the similarity with real life situations. For example, only one game cannot be included in a straight classification of drill and practice game, all the other games, regardless of being a 3D Adventure mini games, a simulator or an action adventure game via Kinect^®^, feature a repetitive training nature. The issue regarding these mini games for drill and practice is that in several studies, it is difficult to differentiate the game mechanics from the outcome neuropsychological test employed.

The intervention protocol used in the referenced studies presented itself relatively homogenous. Our review shows that most of the training protocols employed a minimum of 30 min of video game playing per session (27–30, 32–38), twice a week at least (28, 32, 33, 36, 37), and a wide variation in the duration of the training – all the studies from 3 to 16 weeks. One possible explanation for not performing few and short duration sessions concerns about the smaller statistical power, and, consequently, difficulties in measuring the intervention efficacy. According to the retraining approach (52), the persistent and repetitive practice improves or restores the target skills of interest. Regarding the maintenance phase, only one study reported one monthly boost session during three consecutive months (30). This finding shows the lack of knowledge about the preservation of the training effects, since the maintenance/booster sessions assists the durability and may maximize the gains in the intervention program. Unfortunately, the researchers do not know how often or how many sessions are necessary to have success (22).

In rehabilitation processes, learning transference could occur in very similar contexts (near transfer) and/or in situations that seem remote or dissimilar knowledge (far transfer). Yet, measuring the transfer is very difficult, especially, of cognitive abilities. Shipstead et al. (23) proposes that most of the problems to evaluate near transfer in working memory studies are related to (i) working memory near transfer effects are measured by short-term memory tasks and, (ii) the excessive use of near transfer tasks that closely resemble the method of training. The authors propose that different measures of near transfer need to be used, ones that are distinct from the training task or video game. As concluded by Boot et al. (8), if the game is similar to the test, you are learning the test itself.

### Risk of Bias in Individual Studies

In addition to the analysis, the studies reviewed also underwent systematic scrutiny regarding their methodological features. From this, it was evident that the majority of studies adopted clinical trial designs (13 of 14 studies), but maybe for the cost involved, only six studies were RCTs.

The clinical trials evaluated in the present study showed, in general, adequate methods for participants’ selection. The major problem related to selection process was the use of non-random allocation of participants (i.e., clinical doctors determined the group) or the unclear information related to the allocation processes. Since the grouping is related to the intervention and some studies did not have an active waiting list control group, it was hard to keep the outcome assessors, research personnel, and participants blind to the respective treatment allocation, which is directly related to the high amount of studies with potential performance bias. This knowledge of treatment group allocation directly rises the risk to detect changes in the outcome measures, undermining the intervention finding which was pointed out elsewhere by Boot et al. (8).

A recent study tried to cope with this problem by using not only an active control group, but a non-contact control group, which allows to compare the normal expected changes in performance, thus enhancing the detectability of their study (53). As to the sampling method used, probability-sampling was found to be severely lacking. Therefore, future research should try to carry out research using samples that are more representative. The vast majority of studies presented bias related to the choice of measurement tools and instruments to evaluate outcome. This bias seems to be related to the poor conceptualization of how to evaluate cognitive targets and how these cognitive targets are related to real life situations.

Therefore, some studies use only neuropsychological tests as outcome measure, others employed only behavioral and symptoms scales, which diminishes the observation power of these studies. Boot et al. (8) criticizes studies that employ few measurements to access game change. In our present study, at least three studies employed only one test or scale to evaluate outcomes. On the other side of this coin, Green et al. (54) discusses that the excessive use of scales and tests to measure the same cognitive target could lead to a Type I error, besides this more tests lead to more learning, which reduce the potential to observe transfer from the treatment. Once again, at least four studies employed six or more instruments or large batteries to evaluate cognition. Still within Green et al. (54) comment of the subject, we are not only evaluating the effect of training *x* on ability *y* test by the tool *z* and then by the tool *w*, but, in fact, we are evaluating the effect of training *x* on ability *y* in test *w* after the subject being tested on test *z*. The test *z* affects the performance on test *w* and so on. In short, the main bias present in the reviewed studies can be broadly associated with the following domains: (i) operationalization and measurement issues, (ii) sampling issues, and (iii) performance and change detection issues. It is envisaged by the authors that future research should account for these limitations in order to publish better quality studies in the video game rehabilitation field.

### Cognitive, Behavioral, and Transfer Outcomes

One of the main objectives of the present review was to identify significant cognitive and behavioral changes that have been associated with VGT. Results demonstrated five distinct cognitive abilities gains measured by neuropsychological tests. More specifically, gains in: (i) working memory skills, such as visuospatial and verbal working memory (9, 29, 34, 38, 39) (ii) attention skills such as selective, sustained, divided, vigilance, set-shifting, and response speed. (28, 33), (iii) executive functions, such as, flexibility skills (33) and response inhibition (38, 39), (iv) complex reasoning tasks. (38, 39), (v) short-term memory (verbal and spatial) after the VGT (34). It is reasonable to assume that these changes were affected by the motivation and interest of the participants. In general, the games tend to involve challenging tasks that keep the player’s attention, which contributes to a positive plasticity. Likewise, current games are designed with elements that train intensively these skills, but, most of all, promote the ability to learn, express individual independence and creativity, learn to think systematically and develop social interaction. All of these skills trained in specific situations imposed at each stage of the game are closely linked to functions improved in the studies.

The eight gains related to symptoms and behavior reduction were (i) on both inattentive and hyperactive-impulsive symptoms (27, 28, 30, 32, 38) (ii) in inattentive symptoms only (35, 37) (iii) on hyperactive-impulsive symptoms only (36), (iv) enhanced inhibition and metacognition skills (27), (v) enhanced initiation, planning/organization and working memory (35), (vi) enhanced attentional control (28), (vii) enhanced motivation on task (9), and (viii) gains in academic skills, such as reading comprehension and passage copying (37). Although several studies have reported behavioral improvements, it is important to remember that some parents and teachers were not completely blind and impartial to the experiment, such a study should be. This fact may have influenced the filling of scales, for instance.

In relation to follow-up assessment and generalization and transfer effects, seven studies had a follow-up session to evaluate the maintenance of the gains related to the video game intervention. The time varied from 3 weeks to 6 months after the last training session. Six studies reported maintenance of the training gains in each follow-up assessment session. Only 43% of the studies evaluated transfer effects. Four studies related transfer to non-trained skills such as flexibility (33) and short-term memory (29, 34, 38, 39). Two studies used reports from parents (32) and participants (28) to account for improvements on attentional, organizational and study skills. None of the studies employed any methodology to generalization effects. It was observed that there is a great variation of maintaining the benefits achieved through cognitive training, variables such as the protocol used, the number of sessions and duration of the intervention, individual characteristics of a given population, can influence the support of cognitive and functional gains.

### Summary of Limitations

In the current review, we identified 14 studies that used video games for ADHD intervention. As Bisoglio et al. (42) also highlighted, most of them showed some kind of methodological deficiencies. A significant limitation was the insufficient description of experimental and control conditions, which shows the need for standardization in intervention programs. Some trials did not design a well RCT or adopted a wait-list, placebo, or control training condition (29–31, 34, 36). Another common problem was small sample size (28, 32, 36, 38), the lack of homogeneous group characteristics (9, 32) or the non-standard use of stimulant medication during the rehabilitation. These problems could have affected the statistical power of the trials, thus generating inconsistent results.

An additional factor for consideration is the wide variety of outcome measurements, parent and teacher reporting scales and cognitive tests. Four of the studies we reviewed used just behavior scales: ADHD Rating Scale – ARS-IV, Behavior Rating Inventory of Executive Function – BRIEF, Disruptive Behavior Disorders Rating Scale – DBDRS and Conners’ Rating Scales–Revised – CRS-R (27, 30, 35, 36). Just four studies still applied cognitive tests, from “gold standard” tests as WISC to computerized neuropsychological tasks (9, 34, 38, 39). The other ones associated both tools. On the other hand, it is important to note that video game interventions, usually, did not include functional outcome measures, in other words, everyday functioning and real life VGT benefits are not examined. These analyzed instruments provide information about symptoms, functions and skills, but we do not know if the results are influenced by learning or transfer effects, creating an unclear efficacy of the intervention (13).

There is also a clear lack of information on the follow-up assessments and transfer and generalization effects of training. Ideally, intervention programs should include follow-up assessments, but some studies were not conducted and others emphasized the need of longer-term intervals (9, 29, 31, 35, 37, 38). Unlike other methods, some video games were not designed to improve cognitive or behavior domains, which could be limited for treatments.

Despite the limitations of the studies, the results provide a perspective of the main problems in interventions for ADHD using video games. Overall, innovative and feasible programs are needed as another useful tool in ADHD treatments. As a training tool associated with neuropsychological rehabilitation protocols, video games seem to be essential, both as a motivational and engagement tool and as the main actor in the drill and practice training.

## Conclusion

Attention deficit hyperactivity disorder is a common and chronic childhood disorder with symptoms typically exhibited during elementary school years. It is characterized by difficulties in developing self-control, impairment in academic performance, poor peer and family relationships and psychosocial disadjustment.

As a training tool associated with neuropsychological rehabilitation protocols, video games seem to be essential, both as a motivational and engagement tool and as the main actor in the drill and practice training.

The present review constitutes a relevant step toward understanding the video games as an intervention tool in rehabilitation programs. Considerable advances and new games show us a promising training method for ADHD treatment. However, the lack of blinded assessors and the inappropriate use of scales and tests diminishes the power of these findings.

Despite the positive effects shown by VGT, there are some limitations, such as the variety in the nomenclature, the heterogeneous protocols and methodologically limited literature, which, so far, imposes limits to establish recommended neuropsychological training protocols with video game. More well-designed RCTs with larger samples sizes are necessary to confirm the efficacy of the trials conducted in this area. Other studies will also need to more explicitly clarify the mechanisms associated with training gains, which are hindered, for example, by the use of stimulant drugs during the rehabilitation protocols.

Furthermore, additional researches are important to clarify the effects and stability of the VGT programs, and an important effort should be made to construct better methods to assess improvements on everyday cognitive abilities and real world functioning. The current review provides recommendations and methodological alerts for improving both the process and outcome related issues in research focused on the use of video games in training programs for persons with ADHD.

## Author Contributions

All the authors conceptualized, designed, drafted, and reviewed the paper. Author TR, LN, and EP collected the data and author OB acted to solve any conflict related to the studies selection. All the authors have final approval of the published article and both authors agree to be accountable for all aspects of the work in ensuring that questions related to the accuracy or integrity of any part of the work are appropriately investigated and resolved.

## Conflict of Interest Statement

The authors declare that the research was conducted in the absence of any commercial or financial relationships that could cause a potential conflict of interest.
