# Corrigendum: ADHD Rehabilitation through Video Gaming: A Systematic Review Using PRISMA Guidelines of the Current Findings and the Associated Risk of Bias

**DOI:** 10.3389/fpsyt.2016.00173

**Published:** 2016-10-17

**Authors:** Thiago Strahler Rivero, Lina Maria Herrera Núñez, Emmy Uehara Pires, Orlando Francisco Amodeo Bueno

**Affiliations:** ^1^Grupo de Investigação em Memória Humana, Departamento de Psicobiologia, Universidade Federal de São Paulo, São Paulo, Brazil; ^2^Programa de Psicologia, Universidad Libre, Cali, Colombia; ^3^Departamento de Psicologia, Universidade Federal Rural do Rio de Janeiro, Seropédica, Brazil

**Keywords:** neuropsychological rehabilitation, video game intervention, ADHD, real life, systematic review, PRISMA

Modifications on the original paper are presented below. They were made to address the requests of the researchers whose papers were evaluated in our systematic review. Some corrections refer to mistakes made when data were transferred from tables to text (tables were correct, but some data were mistyped on the text). Other modifications can be classified as more detailed explanations, and they should clarify misunderstandings about the bias analysis or technical video game characteristics. The present corrections does not affect the scientific validity of the results, mainly those associated with the outcomes of the several regimens of videogames training and the possible research bias found throughout the studies that were analyzed. However, we do exalt the importance to associate the article reading with the corrigendum reading, to so fully understand the present findings.

**Page 4, Operationalization of Cognitive Treatment Targets**

This should read

“Five studies had their video game built focusing on working memory (9, 34, 35, 38, 39). One study aimed at inhibitory control abilities (32). Some studies reports combined training. One of them (31) reports a combined working memory and inhibitory control training approach and another study (27) aimed to 3 cognitive targets, working memory, inhibition and cognitive flexibility.”

**Page 4, Operationalization of Video Games Genre**

This should read

“Six studies (27, 29–31, 34, 35) employed 2D or 3D adventure games, this games employs elements of puzzles, exploring, discovering, and other games mechanics related to brain challenges and cognitive skills training. One study employed a gamified WM-task (9). These are the elements that these games employed. It should be clear that the cited works aimed to deal with the process that was the training targets.”

**Page 4, Methodological Features of Studies, 2 paragraph**

This should read

“Ten studies used rating scales for collecting data, but three of them used rating scales only (27, 35, 36), one study (37) associated rating scales and formal education evaluation, four studies (31, 32, 38) used rating scales and cognitive tests methods, one study employed the three above cited tools (29) to assess participants and one study collected rating scale results and also EEG data (30).”

**Page 4, Study Selection and Data Collection Processes**

This should read

“(ii) measurement bias is due to inappropriate use of scales or tests to measure ADHD symptoms and cognitive impairment mainly related to non-validated criteria or inconsistent use (i.e., employing only behavioral scales to measure cognitive change or employed only cognitive tests to measure behavioral changes).”

**Page 10, Limitations Assessments**

This should read

“For example, regarding the difficulties about the study design and method, two mentioned the need for well-designed RCTs (30, 36), two other studies did not adopt a wait-list, a placebo and a control training condition (29, 34) and one did not control game elements, difficulty level and medication (9).”

Final line

“Moreover, variability on some outcome measures (30) and low power differences in teachers’ ratings (27) were also discussed.”

**Page 10, Methodological Features of Studies, 3 paragraph**

This should read

“With respect to rating scales employed in the outcome, one study employed parent evaluation only (37), seven studies used Parent and Teacher rating (27, 29, 30, 32, 35, 36, 38), one study (31) employed parents and other significant adult and one study associated ratings from parents, teachers, clinicians, and the participants (28).”

**Page 10, Limitations Assessments, final line**

This should read

**Page 10, Transfer and Generalization Effects**

This should read

“Six studies did not evaluate transfer effects (9, 27, 31, 34, 35, 37). Four studies related transfer to non-trained skills such as flexibility (33) and working memory (29, 34, 38, 39). Two studies used reports from parents (32) and participants (28) account for improvements on attentional, organizational, and study skills. Two studies (30, 36) used mathematics and English exercise worksheets as a measure of skill transfer. However, the results were not presented in the studies.”

**Page 10, Video Game Protocols Characteristics and Effects of Video Game Intervention on ADHD Participants**

This should read

“The minimum number of training weeks was 3 (9). Six studies trained their participants during 5–6 weeks (27, 29, 31, 35, 38, 39), five studies employed an 8-week regimen of training (28, 30, 33, 37), one study used 6–10 weeks regimen (34), one study used 10 weeks training (36), while another had 16 weeks of intervention (32).”

**Table 1, line 1 (study 27), Cognitive function intervention target,**

This should read

“Inhibition, cognitive flexibility and visualspatial WM”

**Table 2, line 1 (study 27), column 4 (Video Game Type)**

This should read

2D Adventure

**Table 2, line 8, column 4 (Video Game Type)**

This should read

Gamified cognitive task

**Page 11, Risk of Bias in Individual Studies**

This should read

“In terms of selection bias, two studies (9, 35) were rated as high risk due to increased likelihood of bias resulting (…)”

“Detection bias potentially posed high risk in seven studies (27, 28, 30, 32, 35, 36, 37) due to knowledge of the allocated interventions by outcome assessors.”

“Regarding attrition bias, only two studies (31, 32) were rated as high risk for potential attrition bias due to the amount or unclear nature of handling of the missing data. “

“Reporting bias was rated high risk in two studies (36, 37) as some key variables that would have been expected to be reported were not.”

“In addition, measurement bias was judged as high risk in the vast majority of studies (9, 27, 30, 33, 34, 35, 36, 37, 39) due to (i) inconsistent conceptualization of cognitive target, (ii) inconsistent measurement of the outcomes, and/or (iii) inconsistent selection of tools and instruments to evaluate outcome. This kind of bias is related to the lack of an operational definition of a specific cognitive function and its consequent misadministration of an independent evaluation of cognitive targets with tasks recognized by the specialized literature.”

**Page 11, line 5 (study 31), column 2, (Selection bias)**

This should read

unclear risk of bias (?) and not high risk of bias (+)

**Page 11, line 5 (study 31), column 6, (Reporting bias)**

This should read

low risk of bias (−) and not high risk of bias

## Conflict of Interest Statement

The authors declare that the research was conducted in the absence of any commercial or financial relationships that could be construed as a potential conflict of interest.

